# Relationship between estimated desaturase enzyme activity and metabolic syndrome in a longitudinal study

**DOI:** 10.3389/fnut.2022.991277

**Published:** 2022-10-26

**Authors:** Inés Domínguez-López, Camila Arancibia-Riveros, Anna Tresserra-Rimbau, Sara Castro-Barquero, Rosa Casas, Zenaida Vázquez-Ruiz, Emilio Ros, Montserrat Fitó, Ramon Estruch, M. Carmen López-Sabater, Rosa M. Lamuela-Raventós

**Affiliations:** ^1^Department of Nutrition, Food Sciences and Gastronomy, XIA School of Pharmacy and Food Sciences, University of Barcelona, Barcelona, Spain; ^2^INSA-UB, Nutrition and Food Safety Research Institute, University of Barcelona, Santa Coloma de Gramanet, Spain; ^3^CIBER Fisiopatología de la Obesidad y Nutrición (CIBEROBN), Instituto de Salud Carlos III, Madrid, Spain; ^4^Department of Internal Medicine, Hospital Clinic, Institut d’Investigació Biomèdica August Pi i Sunyer (IDIBAPS), University of Barcelona, Barcelona, Spain; ^5^Department of Preventive Medicine and Public Health, University of Navarra, IdiSNA, Pamplona, Spain; ^6^Department of Endocrinology, Hospital Clinic, IDIBAPS, Barcelona, Spain; ^7^Unit of Cardiovascular Risk and Nutrition, Institut Hospital del Mar de Investigaciones Médicas (IMIM), Barcelona, Spain

**Keywords:** fatty acids, desaturases, gas chromatography, PREDIMED, metabolic syndrome, Mediterranean diet

## Abstract

Desaturase enzyme activities (DEA) are associated with several metabolic diseases. The aim of the present study was to assess the relationship between estimated plasma DEA and the metabolic syndrome (MetS), as well as their relationship with individual components of the MetS. We conducted a longitudinal study of 148 participants recruited at random from the PREDIMED trial (Hospital Clinic site). At baseline and after 1 year of follow-up, DEA were estimated from product/precursor ratios of individual plasma fatty acids. Logistic regressions were used to assess the relationship of estimated DEA MetS, adjusted for potential cofounders. Estimated Δ5 desaturase (D5D) activity was associated with lower risk of MetS, whereas stearoyl-CoA (SCD)-16 and SCD-18 were negatively associated with MetS status. SCD-16, SCD-18, and Δ6 desaturase (D6D) were positively associated with triglycerides, SCD-18 was inversely associated with HDL-cholesterol. Estimated D6D activity was found to be associated with increases in diastolic blood pressure. In contrast, D5D was negatively associated with triglycerides, diastolic blood pressure and waist circumference. The present longitudinal study suggests that estimated SCD-16, SCD-18, and D6D have a negative impact in MetS and its components, whereas D5D may have beneficial effects for metabolic health.

## Introduction

The prevalence of metabolic syndrome (MetS) has increased in the last three decades and the global prevalence has been estimated to be about 1 quarter of the world population (1). The prevalence in Spain reached 30% in 2012 (2), and this number is estimated to increase in approximately 94,000 cases every year (3). MetS is defined as a set of criteria that, when grouped together, represent a risk for developing cardiovascular disease (CVD) and type 2 diabetes (T2D), such as elevated blood triglycerides (TG) or glucose (4). The development of MetS can be promoted by unmodifiable risk factors, including genetics or aging, but also by modifiable lifestyle habits, such as physical activity or diet (5). The incidence of MetS is particularly higher in men aged over 45 years with an educational level below university studies. Healthier lifestyle therapies in the management of MetS focus on reducing weight, sedentarism, and improving the diet. It has been reported that the incidence of MetS can be reduced with a higher adherence to the Healthy Lifestyle Score, which includes never smoking, moderate to high physical activity, higher adherence to Mediterranean diet, or moderate alcohol consumption, among others (6). Other studies recommend to reach a greater adherence to Mediterranean diet to reduce its development (3).

The traditional Mediterranean diet has been recognized as protective against the development of MetS and other chronic diseases, such as T2D, CVD, and hypertension (7, 8). This healthy dietary pattern is characterized by a high intake of fruits, vegetables, legumes, nuts, and whole grains, and olive oil (9). The Mediterranean diet provides a high content of healthy fats that mostly come from olive oil and favors a better lipid profile.

**FIGURE 1:**
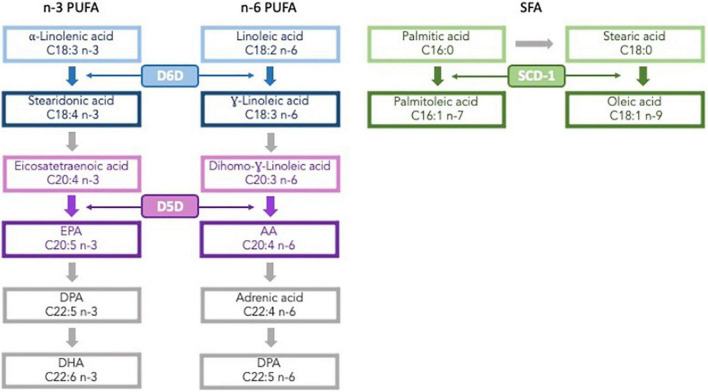
Fatty acid synthetic pathway. PUFA, polyunsaturated fatty acids; D6D, D^6^ desaturase; D5D, D^5^ desaturase; EPA, eicosapentaenoic acid; DPA, docosapentaenoic acid; DHA, docosahexaenoic acid; AA, arachidonic acid; SFA, saturated fatty acids; SCD, stearoyl coenzyme A desaturase.

The plasma fatty acid (FA) profile is considered a more reliable biomarker of dietary fat intake than food frequency questionnaires (FFQ) (10), but it may also be affected by non-dietary factors, such as endogenous metabolism. FAs can be synthesized, elongated, or desaturated in reactions catalyzed by the enzymes stearoyl-CoA desaturase (SCD-1), Δ6 desaturase (D6D), and Δ5 desaturase (D5D) (11), as shown in Figure 1. Altered desaturase enzyme activities (DEA), calculated with the ratios of the FAs that intervene in the reaction, are associated with cardiometabolic risk factors, such as T2D, obesity and MetS (12, 13). However, studies assessing the effect of estimated DEA on MetS and its individual components remain scarce. We hypothesized that altered ratios of estimated DEA would be associated with MetS and the individual components that constitute it. Thus, the aim of this substudy was to assess the relationship of estimated DEA with MetS and its components after 1 year of follow-up in a Mediterranean population.

## Materials and methods

### Study design

The PREDIMED (PREvención con DIeta MEDiterránea) study was a 5-year large, parallel-group, multicenter, randomized, controlled, clinical trial conducted in Spain from October 2003 to December 2010 with the aim of assessing the effect of a Mediterranean diet on the primary prevention of CVD.^1^ In summary, 7,447 participants aged 55–80 years at high cardiovascular risk were included. Eligible participants were men and women with T2D, dyslipidemia, hypertension, overweight/obesity or family history of premature CVD. Exclusion criteria included sever chronic illnesses, alcohol or drug abuse, and BMI > 40 kg/m^2^. A detailed description of methods and participants has been published elsewhere (14).

For the current analysis, we used a randomly selected subsample of participants from the PREDIMED-Hospital Clinic recruitment center. To estimate DEA, a total of 148 participants with available data on plasma FA profiles at baseline and after 1 year of follow-up were included.

The protocol was approved by the Research Ethics Committees at the Hospital Clinic recruiting center and all participants signed a written informed consent form.

### Covariate assessment

A validated semi-quantitative 137-item FFQ was collected by trained dietitians to assess dietary intake at baseline and after 1 year (15). Nutrient intakes were calculated from Spanish food composition tables (16). One female participant who reported implausible energy intakes (>3,500 and <500 Kcal/day for females, and >4,000 and <800 Kcal/day for males) was excluded from the analysis (17). Mediterranean diet adherence was assessed with a 14-item questionnaire with a value of 0 or 1 for each dietary component (18).

Trained personnel carried out anthropometric measurements at baseline and 1-year follow-up. Physical activity was assessed with a validated Spanish version of the Minnesota physical activity questionnaire (19). The anthropometric measurements used in this study were body mass index (BMI), calculated as weight in kg/height^2^ in m^2^, and waist circumference (WC). Diastolic and systolic blood pressure (DBP and SBP, respectively) was measured in triplicate with a validated semi-automatic sphygmomanometer after a minimum of 5 mins rest in a seated position.

### Laboratory measurements

Blood samples were collected after an overnight fast, coded, and stored at –80C° until analysis. Biochemical analyses [glucose, triglycerides, total cholesterol, and high-density lipoprotein cholesterol (HDL-c)] were performed by standard enzymatic procedures. The FA profile in plasma was determined in total lipids by fast gas chromatography with a flame ionization detector (GC-FID) with a previous derivatization to the corresponding FA methyl esters (FAMEs) (20). Fast analyses were performed on a Shimadzu GC-2010 Gas Chromatograph (Shimadzu, Kyoto, Japan) equipped with an FID and Shimadzu AOC-20i Autoinjector. Separation of FAMEs was carried out on a capillary column (10 m × 0.10 mm i.d.), coated with an SGE-BPX70 cross-linked stationary phase (70% cyanopropyl polysilphenylene-siloxane, 0.20 μm film thickness) from SGE (SGE Europe Ltd., United Kingdom). Methyl ester peaks were identified by comparison of their relative retention times with the standards Supelco 37 component FAMEs mix and PUFA No. 2 (Animal source), purchased form Merck (Darmstadt, Germany). Results were expressed as relative percentages of total FAs.

### Estimation of desaturase activities

Plasma FA levels at baseline and changes after 1-year of follow-up are detailed in Supplementary Table 1 according to MetS status.

DEA were estimated as the product/precursor ratios of FAs in plasma according to the following: SCD-16 = C16:1 *n* – 7/C16:0, SCD–18 = C18:1 *n* – 9/C18:0, D6D = C18:3 *n* – 6/C18:2 *n* – 6, and D5D = C20:4 *n* – 6/C20:3 *n* – 6 (11).

### Definition of metabolic syndrome

For the present work we applied the definition of MetS proposed by six major organizations and societies (IDF, NHLBI, AHA, WHF, IAC, and IASO) (21). Accordingly, participants who presented 3 of 5 of the following risk factors were included in the MetS group: elevated TG (>150 mg/dL or drug treatment for elevated TG), reduced HDL-c (< 40 mg/dL in men and <50 mg/dL in women), elevated blood pressure (SBP > 130 and/or DBP > 85 mmHg, or antihypertensive drug treatment), elevated fasting glucose (>100 mg/dL or drug treatment of elevated glucose), and elevated WC (>102 cm for men and >88 for women).

### Statistical analysis

Baseline characteristics of the participants with and without MetS are presented as means ± SD for continuous variables and percentages for categorical variables. *T*-tests were used to assess differences in continuous variables and Chi-Square tests were used for categorical values. *T*-tests were also used to assess differences in plasma FA profile between participants with and without MetS, as well as within-group differences between baseline and 1-year of follow-up.

Baseline values and 1-year changes of estimated DEA were normalized and scaled in multiples of 1-SD with Blom inverse normal transformation (22). Changes in estimated DEA and MetS components were calculated as a 1-year value minus the baseline value.

The associations between the prevalence of MetS and estimated DEA were assessed with a logistic regression analysis to calculate the odds ratios (OR) and 95% confidence interval (CI) adjusting for potential confounders (sex, age, physical activity, BMI, smoking status, educational level, and total energy intake) and stratifying for sex. Multinomial logistic regression was employed to assess the relative risk ratio (RRR) of 1-year changes in MetS status and in estimated DEA, also stratifying for sex, and incorporating the intervention group into the adjustment models.

Multivariable adjusted linear regression models were used to assess differences between estimated DEA per 1-SD increase and MetS components (TG, HDL-c, DBP, SBP, glucose, and WC). The adjustment model for potential confounders included: sex, age, physical activity, smoking status, educational level, total energy intake, and BMI (except for WC). In addition, related medication was added in the adjustment model for each MetS component: TG and HDL-c were further adjusted for cholesterol-lowering drugs; DBP and DSP were further adjusted for antihypertensive medication; glucose was further adjusted for insulin and other hypoglycemic drugs; SCD-16 and SCD-18 were further adjusted for PUFA intake. The longitudinal analysis considering 1-year changes in estimated DEA and MetS components was carried out using the same models, further adjusted for the intervention groups.

For all analyses, two-sided significance was determined at a *p* < 0.05. Analyses were performed with Stata 16.0 (Stata-Corp LP, TX, USA).

## Results

### General characteristics

Table 1 shows the baseline characteristics of the 148 participants according to MetS status. Approximately two thirds of the participants had MetS, whereas 47 volunteers were considered to not suffer from this syndrome. Among the participants with MetS, the majority were women (62.4%), whereas among those without MetS, the majority were men (57.4%). As expected, more participants with MetS suffered from T2D (83.2%), had higher BMI (29.5 ± 3.8 kg/m^2^) and performed less physical activity (249 ± 237.3 METS-min/day). Surprisingly, a higher percentage of participants without MetS had dyslipidaemia (82.98%).

**TABLE 1:** Baseline characteristics of participants according to MetS^1^.

Characteristics	All participants	No MetS (*n* = 47)	MetS (*n* = 101)	*p*-value^2^
Age (years)	66.9 ± 5.9	65.5 ± 6.0	67.5 ± 5.8	0.050
Female (%)	83 (56.1)	20 (42.6)	63 (62.4)	0.024
Weight (kg)	74.1 ± 11.5	71.8 ± 10.5	75.1 ± 11.9	0.106
BMI (kg/m^2^)	28.9 ± 3.7	27.5 ± 3.2	29.5 ± 3.8	0.002
T2D (%)	109 (73.7)	25 (53.2)	84 (83.2)	<0.001
Dyslipidaemia (%)	101 (68.2)	39 (83.0)	62 (61.4)	0.009
Hypertension (%)	96 (64.9)	27 (57.5)	69 (68.3)	0.197
Current smoker (%)	22 (14.9)	5 (10.6)	17 (16.8)	0.527
Education level (%)				0.667
Low	95 (64.2)	29 (61.7)	66 (65.4)	
Medium and high	53 (35.8)	18 (38.3)	35 (34.7)	
Physical activity (METS-min/day)	278.4 ± 256.3	341.0 ± 285.8	249.3 ± 237.3	0.043
Mediterranean adherence, score	3 ± 4	3 ± 4	3 ± 4	0.882

MetS, metabolic syndrome; T2D, type 2 diabetes; METS, metabolic equivalents.

^1^Continuous variables are shown as means ± SDs, and categorical variables are shown as percentages.

^2^*T*-tests were used for continuous variables, and a chi-square test was used for categorical variables.

Dietary intake of all participants and stratified for MetS are in Table 2. The mean energy intake was 2,515.6 ± 541.1 kcal/day and the most consumed type of fat were monounsaturated fatty acids (MUFA). The two groups of participants with and without MetS were well-balanced and there were no differences in any nutrient except for polyunsaturaded fatty acids (PUFA) intake, as those with MetS reported significantly lower consumption (17.1 ± 6.6 g/day).

**TABLE 2:** Dietary intake for all participants and according to MetS at baseline^1^.

	All participants	No MetS (*n* = 47)	MetS (*n* = 101)	*p*-value^2^
Energy (kcal/day)	2515.6 ± 541.1	2633.0 ± 470.3	2461.0 ± 564.9	0.072
Carbohydrates (g/day)	273.9 ± 80.3	288.96 ± 77.7	266.0 ± 81.0	0.121
Proteins (g/day)	105.1 ± 20.6	108.9 ± 22.4	103.3 ± 19.6	0.122
Total fat (g/day)	103.8 ± 26.3	107.3 ± 21.8	102.2 ± 28.1	0.271
SFA (g/day)	28.0 ± 8.6	29.5 ± 8.7	27.3 ± 8.4	0.139
MUFA (g/day)	49.4 ± 13.6	50.1 ± 11.2	49.0 ± 14.6	0.663
PUFA (g/day)	17.9 ± 7.0	19.5 ± 7.6	17.1 ± 6.6	0.047
Cholesterol (mg/day)	395.4 ± 104.6	415.9 ± 109.8	385.8 ± 101.3	0.104
Fiber (g/day)	30.4 ± 8.0	31.6 ± 8.1	29.9 ± 7.9	0.225
Alcohol (g/day)	9.3 ± 13.9	10.8 ± 16.9	8.6 ± 12.3	0.373

SFA, saturated fatty acids; MUFA, monounsaturated fatty acids; PUFA, polyunsaturated fatty acids.

^1^Variables are shown as means ± SDs.

^2^Differences between groups calculated by *T*-tests.

### Associations of desaturase activities and metabolic syndrome at baseline

The OR of having MetS according to estimated DEA per 1-SD increase at baseline are shown in Table 3. The logistic regression model showed that in all participants higher rates of D5D activity were associated with lower incidence of MetS [OR = 0.53 (95% CI: 0.36; 0.79), *p*-value = 0.002]. Higher SCD-18 activity tended to lower the incidence of MetS, although it did not achieve statistical significance [OR = 1.54 (95% CI: 0.96; 2.58), *p*-value = 0.096].

**TABLE 3:** Odds ratio associated with having MetS with DEA according to sex at baseline per 1-SD increase (*n* = 148).

	OR (CI 95%)	*p*-value^1^
SCD-16	1.08 (0.73; 1.62)	0.691
SCD-18	1.54 (0.96; 2.58)	0.096
D6D	1.18 (0.80; 1.73)	0.397
D5D	0.53 (0.36; 0.79)	0.002

MetS, metabolic syndrome; DEA, desaturase enzyme activities; SCD, stearoyl-CoA desaturase; D6D, D^6^ desaturase; D5D, D^5^ desaturase. Odds ratio (95% confidence interval) by logistic regression analysis. *p* < 0.05.

^1^Adjusted for age, sex, smoking status, educational level, BMI, physical activity, and total energy intake. SCD-16 and SCD-18 were further adjusted for PUFA intake.

The associations between estimated DEA per 1-SD and MetS components after full adjustment are shown in Table 4. Higher estimated SCD-18 and D6D activity were positively associated with higher concentrations of TG [β = 40.50 (95% CI: 23.00; 58.02) per 1-SD increase, *p*-value = 0.001, and β = 15.45 (95% CI: 1.57; 29.33) per 1-SD increase, *p*-value = 0.029, respectively]. Higher rates of estimated SCD-18 activity were also associated with lower HDL-c [β = –4.02 (95% CI: –6.20; –1.85) per 1-SD increase, *p*-value = < 0.001]. Lastly, DBP and WC were inversely associated with estimated D5D activity [β = –1.79 (95% CI: –3.10; 0.48) per 1-SD increase, *p*-value = 0.008, and β = –0.94 (95% CI: –1.82; 0.06) per 1-SD increase, *p*-value = 0.037].

**TABLE 4:** Multivariable-adjusted regression for estimated DEA per 1-SD increase and MetS components at baseline.

SCD-16		SCD-18		D6D		D5D	
β (CI 95%)	*p*-value	β (CI 95%)	*p*-value	β (CI 95%)	*p*-value	β (CI 95%)	*p*-value
**TG^1^**							
14.41 (–0.49; 29.31)	0.058	40.50 (23.00; 58.02)	< *0*.001	15.45 (1.57; 29.33)	0.029	–11.62 (–30.38; 7.13)	0.223
**HDL-c^1^**							
0.68 (–1.68; 3.04)	0.570	–4.02 (–6.20; –1.85)	< *0*.001	–0.66 (–2.91; 1.60)	0.565	0.85 (–1.19; 2.90)	0.410
**DBP^2^**							
1.12 (–0.34; 2.58)	0.131	0.96 (–0.51; 2.44)	0.198	1.18 (–0.51; 2.88)	0.170	–1.79 (–3.10; 0.48)	0.008
**SBP^2^**							
0.05 (–3.17; 3.26)	0.976	0.71 (–1.97; 3.38)	0.602	2.05 (–0.67; 4.78)	0.138	–2.16 (–4.82; 0.49)	0.109
**Glucose^3^**							
4.02 (–2.31; 10.36)	0.211	7.37 (–0.47; 15.20)	0.065	6.24 (–2.17; 14.65)	0.145	4.39 (–1.21; 9.99)	0.123
**WC^4^**							
0.17 (–0.74; 1.09)	0.711	0.01 (–0.94; 0.95)	0.986	–0.03 (–0.96; 0.89)	0.944	–0.94 (–1.82; -0.06)	0.037

MetS, metabolic syndrome; DEA, desaturase enzyme activities; SCD, stearoyl**-**CoA desaturase; D6D, D^6^ desaturase; D5D, D^5^ desaturase; TG, triglycerides; HDL-c, high-density lipoprotein cholesterol; DBP, diastolic blood pressure; SBP, systolic blood pressure; WC, waist circumference; ß, difference between groups; CI, confidence interval.

^1^Adjusted for age, sex, smoking status, educational level, BMI, physical activity, total energy intake, and cholesterol-lowering medication. SCD-16 and SCD-18 were further adjusted for PUFA intake.

^2^Adjusted for age, sex, smoking status, educational level, BMI, physical activity, total energy intake, and antihypertensive medication. SCD-16 and SCD-18 were further adjusted for PUFA intake.

^3^Adjusted for age, sex, smoking status, educational level, BMI, physical activity, total energy intake, insulin and hypoglycemic medication. SCD-16 and SCD-18 were further adjusted for PUFA intake.

^4^Adjusted for age, sex, smoking status, educational level, physical activity, and total energy intake. SCD-16 and SCD-18 were further adjusted for PUFA intake.

### Associations of desaturase activities and metabolic syndrome after 1 year of follow-up

The relationship between 1-year changes in MetS status and estimated DEA per 1-SD increase is presented in Table 5. The multinomial regression model showed that increases in estimated D5D activity were associated with MetS improvement [OR = 2.04 (95% CI: 1.18; 3.56), *p*-value = 0.011], whereas increases in estimated SCD-18 activity were associated with a lower probability of improving MetS status [OR = 0.46 (95% CI: 0.29; 0.71), *p*-value = 0.001]. In addition, higher estimated SCD-16 activity was associated with an increased risk of worsening MetS status [OR = 2.15 (95% CI: 1.18; 3.94), *p*-value = 0.013].

**TABLE 5:** Multinomial logistic regression for changes in MetS status and estimated DEA ratios after 1 year of follow-up per 1-SD increase.

	Maintained MetS status	MetS status improvement		MetS status worsening	
		RRR (CI 95%)	*p*-value^1^	RRR (CI 95%)	*p*-value^1^
SCD-16	Ref.	0.78 (0.46; 1.33)	0.365	2.15 (1.18; 3.94)	0.013
SCD-18	Ref.	0.46 (0.29; 0.71)	0.001	0.63 (0.35; 1.16)	0.136
D6D	Ref.	0.98 (0.66; 1.45)	0.923	1.23 (0.71; 2.11)	0.462
D5D	Ref.	2.04 (1.18; 3.56)	0.011	0.76 (0.33; 1.78)	0.535

MetS, metabolic syndrome; DEA, desaturase enzyme activities; SCD, stearoyl-CoA desaturase; D6D, D^6^ desaturase; D5D, D^5^ desaturase. Relative risk ratio (95% confidence interval) by multinomial logistic analysis. *p* < 0.05.

^1^Adjusted for age, sex, smoking status, educational level, BMI, physical activity, total energy intake, and intervention group. SCD-16 and SCD-18 were further adjusted for PUFA intake.

Table 6 shows the associations between 1-year changes in DEA per 1-SD increase and MetS components. Changes in estimated SCD-16 and SCD-18 activity were positively associated with higher TG [β = 18.08 (95% CI: 7.02; 29.13) per 1-SD increase, *p*-value = 0.002, and β = 34.88 (95% CI: 19.97; 49.79) per 1-SD increase, *p*-value = 0.001, respectively]. Increases in the rate of estimated SCD-18 were associated with decreases in HDL-c concentrations [β = –1.53 (95% CI: –2.62; –0.44) per 1-SD increase, *p*-value = 0.006], whereas increases in levels of estimated D6D activity were associated with higher DBP [β = 1.70 (95% CI: 0.44; 2.97) per 1-SD increase, *p*-value = 0.009]. Finally, we observed a decrease in TG and DBP when estimated D5D activity increased [β = –13.09 (95% CI: –24.99; –1.19) per 1-SD increase, *p*-value = 0.031, and β = –1.52 (95% CI: –2.78; –0.26) per 1-SD increase, *p*-value = 0.018, respectively].

**TABLE 6:** Multivariable-adjusted regression for 1-year changes in estimated DEA per 1-SD increase and MetS components.

SCD-16		SCD-18		D6D		D5D	
β (CI 95%)	*p*-value	β (CI 95%)	*p*-value	β (CI 95%)	*p*-value	β (CI 95%)	*p*-value
**TG^1^**							
18.08 (7.02; 29.13)	0.002	34.88 (19.97; 49.79)	< *0*.001	1.71 (–11.35; 14.76)	0.796	–13.09 (–24.99; –1.19)	0.031
**HDL-c^1^**							
–0.85 (–2.40; 0.71)	0.282	–1.53 (–2.62; –0.44)	0.006	–0.19 (–1.49; 1.11)	0.776	–0.45 (–1.70; 0.80)	0.477
**DBP^2^**							
1.16 (–0.02; 2.35)	0.055	0.68 (–0.83; 2.19)	0.374	1.70 (–0.44; 2.97)	0.009	–1.52 (–2.78; –0.26)	0.018
**SBP^2^**							
0.68 (–1.49; 2.85)	0.537	0.03 (–2.50; 2.55)	0.984	1.52 (–0.51; 3.56)	0.141	–2.25 (–4.64; 0.13)	0.064
**Glucose^3^**							
2.41 (–4.97; 9.78)	0.520	1.00 (–5.17; 7.17)	0.750	1.99 (–4.55; 8.53)	0.548	–1.80 (–8.84; 5.23)	0.613
**WC^4^**							
0.56 (–0.36; 1.48)	0.234	1.28 (0.40; 2.17)	0.005	0.12 (–0.75; 1.00)	0.783	–0.59 (–1.46; 0.28)	0.181

MetS, metabolic syndrome; DEA, desaturase enzyme activities; SCD, stearoyl coenzyme A desaturase; D6D, D^6^ desaturase; D5D, D^5^ desaturase; TG, triglycerides; HDL-c, high-density lipoprotein cholesterol; DBP, diastolic blood pressure; SBP, systolic blood pressure; WC, waist circumference; ß, difference between groups; CI, confidence interval.

^1^Adjusted for age, sex, smoking status, educational level, BMI, physical activity, total energy intake, intervention group, and cholesterol-lowering medication. SCD-16 and SCD-18 were further adjusted for PUFA intake.

^2^Adjusted for age, sex, smoking status, educational level, BMI, physical activity, total energy intake, intervention group, and antihypertensive medication. SCD-16 and SCD-18 were further adjusted for PUFA intake.

^3^Adjusted for age, sex, smoking status, educational level, BMI, physical activity, total energy intake, intervention group, insulin, and hypoglycemic medication. SCD-16 and SCD-18 were further adjusted for PUFA intake.

^4^Adjusted for age, sex, smoking status, educational level, physical activity, total energy intake, and intervention group. SCD-16 and SCD-18 were further adjusted for PUFA intake.

## Discussion

In the present longitudinal substudy of the PREDIMED trial, we observed that higher estimated activities of SCD-16, SCD18, and D6D had an adverse effect on MetS status and its components after 1 year of follow-up. In contrast, estimated D5D activity showed a protective effect against MetS and its components, particularly TG and DBP. To our knowledge, this is the first study to assess the effect of estimated DEA with MetS and its components after 1 year of follow-up in a Mediterranean population.

The activity of these desaturases is known to be related to metabolic health. Differences in the plasma FA profile and estimated DEA have been previously described between metabolically healthy and unhealthy individuals (23, 24). On this basis, Svendsen et al. proposed that these enzymatic activities could serve as novel biomarkers of metabolic health (13, 25). This is in accordance with the results of the present study, as we found that estimated DEA were associated with MetS at baseline and after 1 year of follow-up.

The analysis of plasma estimated DEA related to FA metabolism showed a beneficial effect of estimated D5D activity on the prevalence of MetS. These results are consistent with previous studies that report a positive influence of D5D on cardiovascular health. For example, higher D5D activity has been favorably associated with stroke risk factors, T2D, and abdominal obesity (26–28). D5D is the rate-limiting enzyme that catalyzes the transformation of eicosatetraenoic and dihomo-gamma-linoleic acid into eicosapentaenoic acid (EPA) and arachidonic acid (AA), respectively. Therefore, lower D5D activity leads to the accumulation of precursors and other intermediate FAs that increase cardiometabolic risk, such as gamma-linoleic or dihomo-gamma-linoleic acid (29). Despite all these findings, Mayneris-Perxachs et al. did not observe any association between D5D and the odds of having MetS in a cross-sectional sub-analysis with baseline data of the PREDIMED study (30). However, they did find that D6D and SCD-18 were adversely associated with MetS, which is consistent with our results. The activity of D6D, the key enzyme in the conversion of linoleic and alpha-linoleic acid, and SCD-1, which catalyzes the synthesis of MUFA from saturated fatty acids (SFA), is inhibited by PUFA intake (31). In this regard, evidence obtained in clinical trials has shown that diets with high intakes of PUFA down-regulate SCD-1 activity (32), particularly PUFA resulting from fish consumption (33). However, in relation with our findings, including PUFA intake as a confounder variable in the analyses of SCD-16 and SCD-18 minimally altered the results, suggesting that their associations with MetS and its components were not dependent on PUFA intake. Overall, our findings confirm the results of previous studies in which elevated D5D and reduced D6D and SCD-1 activities positively impacted cardiometabolic risk factors (24, 34).

SCD-16, SCD-18, and D6D are generally known to exert negative effects on metabolic health and other CVD risk factors. SCD-16 and SCD-18 have also been positively associated with BMI, blood pressure, and total cholesterol (35, 36), which is in accordance with our results. Other studies have found that D6D is related to higher TG, blood pressure, BMI and total cholesterol (37, 38). Moreover, D6D has showed a positive association with inflammatory biomarkers, such as ICAM-1 or C-reactive protein (37, 39), which suggests that this enzyme has a negative effect on metabolic health due to the activation of inflammatory pathways.

In contrast, estimated D5D activity has been favorably associated with MetS components, as it has been to related to higher HDL-c, lower blood pressure, and lower BMI (36, 40). Several mechanisms may explain the associations found between estimated DEA and MetS components. PUFA synthesized by D5D and D6D can modulate the expression of transcription factors that participate in lipogenesis and FA oxidation, such as PPAR. In addition, these FA also produce eicosanoids, which are inflammatory mediators that play major roles in lipogenesis or insulin resistance (41). Taken together, these results suggest that D5D products are involved in antiinflammatory responses and upregulation of transcription factors that lead to a better lipid profile and decreased CVD risk, whereas D6D and SCD products may have the opposite effect.

The main strength of the present study is its longitudinal nature, as this is considered the most rigorous method to establish a cause-effect relationship. Other strengths include the analysis of biological samples. Among the limitations of the study is that all the participants were >55 years and at high risk of CVD, thus the results may not be representative of other populations. Additionally, the sample size was relatively small compared to similar studies.

The present study shows that in a Mediterranean population of over 55 years and at high cardiovascular risk, estimated SCD-16, SCD-18, and D6D activities were adversely associated with MetS, whereas D5D was associated with a protective effect. Among the components that constitute the MetS, TG, HDL-c, DBP, and WC were adversely affected by estimated activities of SCD-16, SCD-18, and D6D. In contrast, D5D was associated with beneficial changes in TG and DBP. Therefore, our results contribute to the hypothesis that FA metabolism influences metabolic health and desaturases dysregulations may be indicative of metabolic alterations. Further research is needed to confirm the current findings in the general population.

## Data availability statement

The data analyzed in this study is subject to the following licenses/restrictions: The datasets presented in this article are not readily available because there are restrictions on the availability of data for the PREDIMED trial, due to the signed consent agreements around data sharing. Requestors wishing to access the PREDIMED-dataset generated and/or analyzed during the current study can make a request to the PREDIMED trial Steering Committee chair. Requests to access these datasets should be directed to RL-R, lamuela@ub.edu.

## Ethics statement

The studies involving human participants were reviewed and approved by the Research Ethics Committees at the Hospital Clinic recruiting center and all participants signed a written informed consent form. The patients/participants provided their written informed consent to participate in this study.

## Author contributions

ID-L: conceptualization, investigation, formal analysis, and writing – original draft. CA-R: methodology and writing – original draft. AT-R, RC, and ZV-R: writing – review and editing. SC-B: methodology and writing – review and editing. ER, MF, and RE: investigation and writing – review and editing. ML-S: formal analysis and writing – review and editing. RL-R: conceptualization, investigation, and writing – review and editing. All authors contributed to the article and approved the submitted version.

## Footnotes

1
http://www.predimed.es

## Abbreviations

AA: arachidonic acid
BMI: body mass index
CVD: cardiovascular disease
DBP: diastolic blood pressure
D5D: Δ 5 desaturase
D6D: Δ 6 desaturase
DEA: desaturase enzyme activities
EPA: eicosapentaenoic acid
FA: fatty acids
FAME: fatty acid methyl ester
FID: flame ionization detector
FFQ: food frequency questionnaire
GC: gas chromatography
HDL-c: high-density lipoprotein cholesterol
MetS: metabolic syndrome
MUFA: monounsaturated fatty acid
PUFA: polyunsaturated fatty acid
SFA: saturated fatty acids
SCD: stearoyl-CoA desaturase
SBP: systolic blood pressure
TG: triglycerides
T2D: type 2 diabetes.
