# Supplementary material for: Relationship between estimated desaturase enzyme activity and metabolic syndrome in a longitudinal study
Source: Front Nutr. 2022 Oct 26;9:991277. doi: 10.3389/fnut.2022.991277 (PMC9643862; doi:10.3389/fnut.2022.991277)
Supplement: Supplementary file 1 [file Table_1.DOCX]

**Supplementary Material**

| **Table S1**. Plasma fatty acid levels at baseline and 1-year changes according to MetS status | | | | | | |
| --- | --- | --- | --- | --- | --- | --- |
|  |  | Mean + SD | *p*-value^1^ | Mean + SD | *p*-value^1^ | *p-*value^2^ |
| C14:0 | Baseline | 0.57 + 0.18 |  | 0.77 + 0.28 |  |  |
|  | Change | -0.01 + 0.19 | 0.748 | -0.02 + 0.247 | 0.469 | <0.001 |
| C16:0 | Baseline | 21.19 + 1.54 |  | 23.01 + 2.26 |  |  |
|  | Change | 0.04 + 1.38 | 0.842 | -0.08 + 2.00 | 0.705 | <0.001 |
| C16:1 n-7 | Baseline | 1.21 + 0.41 |  | 1.49 + 0.61 |  |  |
|  | Change | -0.05 + 0.34 | 0.284 | -0.06 + 0.35 | 0.073 | 0.004 |
| C18:0 | Baseline | 6.54 + 0.61 |  | 6.38 + 0.61 |  |  |
|  | Change | 0.08 + -0.48 | 0.237 | 0.03 + 0.56 | 0.552 | 0.150 |
| C18:1 n-9 | Baseline | 26.47 + 5.05 |  | 28.40 + 4.35 |  |  |
|  | Change | 0.07 + 4.03 | 0.904 | -0.29 + 3.83 | 0.442 | 0.019 |
| C18:2 n-6 | Baseline | 31.01 + 5.00 |  | 28.13 + 5.45 |  |  |
|  | Change | 0.17 + 4.12 | 0.773 | -0.09 + 4.22 | 0.823 | 0.003 |
| C18:3 n-6 | Baseline | 0.38 + 0.17 |  | 0.42 + 0.18 |  |  |
|  | Change | 0.02 + 0.11 | 0.234 | 0.01 + 0.12 | 0.429 | 0.263 |
| C18:3 n-3 | Baseline | 0.29 + 0.12 |  | 0.36 + 0.20 |  |  |
|  | Change | 0.07 + 0.16 | 0.008 | <0.01 + 0.25 | 0.869 | 0.031 |
| C20:3 n-6 | Baseline | 1.40 + 0.31 |  | 1.49 + 0.36 |  |  |
|  | Change | 0.01 + 0.23 | 0.800 | 0.01 + 0.27 | 0.761 | 0.174 |
| C20:4 n-6 | Baseline | 7.06 + 1.35 |  | 6.21 + 1.66 |  |  |
|  | Change | -0.31 + 0.97 | 0.033 | 0.21 + 1.07 | 0.051 | 0.003 |
| C20:5 n-3 | Baseline | 0.94 + 0.70 |  | 0.75 + 0.46 |  |  |
|  | Change | 0.01 + 0.52 | 0.883 | 0.12 + 0.54 | 0.030 | 0.058 |
| C22:6 n-3 | Baseline | 2.75 + 0.84 |  | 2.39 + 0.68 |  |  |
|  | Change | -0.11 + 0.65 | 0.266 | 0.16 + 0.66 | 0.016 | 0.006 |
| MetS, Metabolic Syndrome.  Values are expressed as geometric mean (% of total fatty acids) + SD.  *p*-value^1^ for within-group differences from baseline by paired T-test.  *p*-value^2^ for between-group differences from baseline by paired T-test. | | | | | | |
